# Profound analgesia is associated with a truncated peptide resulting from tissue specific alternative splicing of DRG CA8-204 regulated by an exon-level *cis*-eQTL

**DOI:** 10.1371/journal.pgen.1008226

**Published:** 2019-06-14

**Authors:** Udita Upadhyay, Gerald Z. Zhuang, Luda Diatchenko, Marc Parisien, Yuan Kang, Konstantinos D. Sarantopoulos, Eden R. Martin, Shad B. Smith, William Maixner, Roy C. Levitt

**Affiliations:** 1 Department of Anesthesiology, Perioperative Medicine and Pain Management, University of Miami Miller School of Medicine, Miami, Florida, United States of America; 2 Alan Edwards Centre for Research on Pain, McGill University Department of Anesthesiology, Montreal, Quebec, Canada; 3 Bascom Palmer Eye Institute, University of Miami Miller School of Medicine, Miami, Florida, United States of America; 4 John T. MacDonald Foundation Department of Human Genetics, University of Miami Miller School of Medicine, Miami, Florida, United States of America; 5 John P. Hussman Institute for Human Genomics, University of Miami Miller School of Medicine, Miami, Florida, United States of America; 6 Center for Translational Pain Medicine, Department of Anesthesiology, Duke University School of Medicine, Durham, North Carolina, United States of America; Boston Children’s Hospital and Harvard Medical School, UNITED STATES

## Abstract

Carbonic anhydrase-8 (CA8) is an intracellular protein that functions as an allosteric inhibitor of inositol trisphosphate receptor-1 (ITPR1) critical to intracellular Ca^++^ release, synaptic functions and neuronal excitability. We showed previously that murine nociception and analgesic responses are regulated by the expression of this gene in dorsal root ganglion (DRG) associated with a *cis*-eQTL. In this report, we identify an exon-level *cis*-eQTL (rs6471859) that regulates human DRG CA8 alternative splicing, producing a truncated 1,697bp transcript (e.g., CA8-204). Our functional genomic studies show the “G” allele at rs6471859 produces a cryptic 3’UTR splice site regulating expression of CA8-204. We developed constructs to study the expression and function of the naturally occurring CA8-204^G^ transcript (G allele at rs6471859), CA8-204^C^ (C allele at rs6471859 reversion mutation) and CA8-201 (full length transcript). CA8-204^G^ transcript expression occurred predominantly in non-neuronal cells (HEK293), while CA8-204^C^ expression was restricted to neuronal derived cells (NBL) *in vitro*. CA8-204^G^ produced a stable truncated transcript in HEK293 cells that was barely detectable in NBL cells. We also show CA8-204 produces a stable peptide that inhibits pITPR1 and Ca^++^ release in HEK293 cells. These results imply homozygous G/G individuals at rs6471859, which are common in the general population, produce exclusively CA8-204^G^ that is barely detectable in neuronal cells. CA8 null mutations that greatly impact neuronal functions are associated with severe forms of spinal cerebellar ataxia, and our data suggest G/G homozygotes should display a similar phenotype. To address this question, we show *in vivo* using AAV8-FLAG-CA8-204^G^ and AAV8-V5-CA8-201 gene transfer delivered via intra-neural sciatic nerve injection (SN), that these viral constructs are able to transduce DRG cells and produce similar analgesic and anti-hyperalgesic responses to inflammatory pain. Immunohistochemistry (IHC) examinations of DRG tissues further show CA8-204^G^ peptide is expressed in advillin expressing neuronal cells, but to a lesser extent compared to glial cells. These findings explain why G/G homozygotes that exclusively produce this truncated functional peptide in DRG evade a severe phenotype. These genomic studies significantly advance the literature regarding structure-function studies on CA8-ITPR1 critical to calcium signaling pathways, synaptic functioning, neuronal excitability and analgesic responses.

## Author summary

Carbonic anhydrase-8 (CA8) inhibits IP3 binding to the inositol trisphosphate receptor-1(ITPR1), which regulates intracellular calcium signaling critical to neuronal functions. Recessive CA8-null mutants are associated with spinocerebellar ataxia and neurodegenerative disorders. We have previously demonstrated that nociception and analgesic responses are associated with a DRG *cis*-eQTL that regulates murine expression of this gene. This study focuses on a human DRG exon-level *cis*-eQTL (rs6471859) that regulates CA8-204 alternative splicing producing a truncated 1,697bp transcript. Herein, we demonstrate the “G” allele at rs6471859 produces a cryptic 3’UTR splice site regulating tissue-specific CA8-204 expression. *In vitro* studies show the “G” allele (CA8-204^G^) produces a stable peptide that inhibits ITPR1 activation and Ca^++^ release in non-neuronal cells, but not in neuronal cells. However, using AAV8 gene transfer *in vivo* we show CA8-204^G^ peptide is expressed in both glial and to a lesser extent in neuronal cells, producing profound analgesia and anti-hyperalgesia using inflammatory pain models, similar to the full length CA8-201 positive control. These data significantly extend our understanding of CA8 structure-function, demonstrating the truncated peptide may represent a novel therapeutic candidate.

## Introduction

Car8 and its human ortholog (CA8) are enzymatically inactive isoforms of carbonic anhydrase that inhibit activation of neuronal inositol 1,4,5-trisphosphate receptor type-1 (ITPR1) by phosphorylation (pITPR1), and intracellular calcium release in mice. ITPR1 converts inositol trisphosphate (IP3) signaling to intracellular calcium signaling [1], essential to the regulation of neuronal excitability, synaptic morphology [2]. Previously, Hirota *et al*., (2003) determined essentially all 8 exons of Car8 (amino acids 45–291 of Car8-201) are minimally required for the direct binding to the modulatory domain of ITPR1 (amino acids 1,387–1,647) [3]. Furthermore, they showed Car8 acts as an allosteric inhibitor of IP3 binding to ITPR1, thereby inhibiting calcium signaling. Humans with a CA8 null mutation occurring in exon three (S100P), demonstrate a severe form of spinal cerebellar ataxia associated with quadrupedal locomotion and mental retardation; further defining a critical role for CA8 in neuronal development and functioning [4].

Previously, we reported Car8/CA8 and its role in calcium signaling are critical to nociception, inflammatory and neuropathic pain [5, 6]. Additionally, we reported Car8/CA8 gene therapy was analgesic and anti-hyperalgesic in animal models of pain [5, 6]. In order to quantify analgesic responses observed in animal models after Car8/CA8 gene therapy, we used allometric conversions indicating profound analgesia that exceeded an oral dose of 250 mg of morphine in an average sized adult [7]. Using integrative genomics to understand the impact of Car8 genetic regulation on nociceptive responses we identified a murine gene-level *cis-*expression quantitative trait (eQTL) in DRG associated with variable *Car8* gene expression. This *Car8* DRG *cis*-eQTL was shown to regulate nociception and analgesic responses [8].

In this report, we identify a 3’UTR single nucleotide polymorphism (SNP)(rs6471859) that functions as an exon-level *cis*-eQTL in human DRG. Through our functional genomic studies, we found the “G” allele to be associated with probe PSR0801622.hg1 (P = 0.0095) exclusively recognizing a truncated 1,697 bp transcript (e.g., CA8-204). CA8-204 comprises coding sequence for exons 1–3 with a retained intron with a self-contained polyadenylation signal and coding for an extended exon 3 associated with a new stop. It is believed that CA8-204 is non-coding in current public databases. Based on the work of Hirota *et al*., (2003) this truncated peptide is not expected to bind ITPR1 directly, impact IP3 binding, nor influence calcium signaling [3]. We also report that this eQTL directs tissue-specific alternative splicing, where CA8-204 is predominantly expressed in non-neuronal cells *in vitro*. Additionally, 1000 Genomes Project Phase 3 data show G/G homozygous individuals at rs6471859 are prevalent in the general population, with their frequencies differing by ethnic background. While the G allele is on average more prevalent in populations of East Asian ancestry (G: 75%; G/G homozygotes 56%); and less prevalent on average in populations of African ancestry (G: 15%; G/G homozygotes 2%); there is a near equal average prevalence in populations of European ancestry (G: 58%; G/G homozygotes 34%). Thus, G/G homozygotes are common in the general population and are expected to exclusively express the no-coding CA8-204 transcript predominantly in non-neuronal DRG cells. Given CA8 deficiency is associated with severe spinal cerebellar ataxia and that we have reported CA8 is critical to nociception, inflammatory and neuropathic pain [5, 6], neuronal DRG wildtype CA8 (transcript CA8-201) deficiency associated with G/G genotype should display a deleterious phenotype. Therefore, it was imperative that we clarify for the literature the significance of this *cis-*eQTL on DRG CA8 tissue-specific alternative splicing, and the role of CA8-204 in calcium signaling, nociception and analgesic responses.

Herein using QPCR, we show that rs6471859 regulates tissue-specific alternative splicing of CA8-204. Specifically, *in vitro* we found that the ‘G’ allele produces a cryptic splice site regulating expression of this transcript primarily in non-neuronal HEK293 cells. We also determined that CA8-204 codes for a truncated protein product, which inhibits steady state pITPR1, much like wild type CA8 (CA8-201). Similarly, like CA8-201, this truncated CA8-204 peptide also negatively regulates intracellular calcium release *in vitro*. Finally, using gene transfer containing AAV8-CA8-204 viral particles *in vivo* we show this peptide is expressed in both neuronal and glial cells within transduced DRG producing analgesia and anti-hyperalgesia. Collectively, these studies explain why G/G homozygotes at rs6471859 that exclusively produce this truncated peptide in DRG evade a severe phenotype. These genomic studies significantly advance the literature regarding structure-function studies on CA8-ITPR1 critical to calcium signaling pathways, synaptic functioning, neuronal excitability and analgesic responses.

## Results

### Identification of a human DRG exon-level *cis*-eQTL associated with CA8-204 expression in a tissue-specific manner

**Fig 1:**
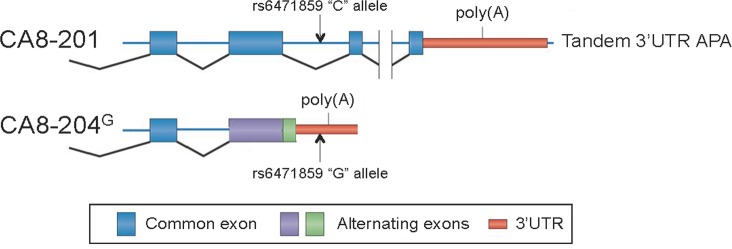
Illustration of CA8-204 alternative splicing with a retained intron harboring *cis*-eQTL rs6471859 within a retained intron, extended exon 3 and a self-contained polyadenylation site. A graphic representation of exon allocation for CA8-201 and alternatively spliced CA8-204^G^. CA8-204 harbors a cryptic splice site in the 3’UTR produced by the “G” allele at rs6471859 acting as an eQTL to produce tissue-specific alternative splicing (APA = Alternative polyadenylation).

**Table 1** shows the association between rs6471859 with exon-level probe PSR08016202.hg1 (P = 0.0095) regulating expression of alternative transcript CA8-204. CA8-204 is comprised of the first three exons of CA8-201 with an extended exon 3 due to a retained intron containing a new stop (**Fig 1**). These data suggest that rs6471859 represents an exon-level *cis*-eQTL that resides at nucleotide position 1,416 within the retained intron of the CA8-204 transcript regulating expression of this alternatively spliced transcript in DRG. The exact mechanism by which rs6471859 regulates the alternative splicing of CA8-204 remains unknown.

**Table 1:** SNP rs6471859 is associated with probe recognizing CA8-204, representing a potential *cis*-eQTL. rs6471859 regulates the alternative splicing of CA8-204. rs6471859 was identified as a DRG exon-level *cis*-eQTL associated with probe PSR0801622.hg1 (P = 0.0095) identifying exclusively the alternatively spliced CA8-204 transcript. (MAF = minor allele frequency; AVGLVL = average expression value, PVAL = P-value for association. The adjusted significance level was P<0.0125 based on testing for association with four known transcripts. (Note: the effect allele differs depending on the tissue tested and the predominant cell type(s) (Geo accession: GSE78150, NCBI).

SNP	BP	A1	A2	MAF_A1 _Global	Probe	AVGLVL	BETA	TSTAT	**PVAL**
rs6471859	6117762	C	G	0.407	PSR08016202.hg.1	3.71	-0.07	-2.62	0.0095

**Fig 2:**
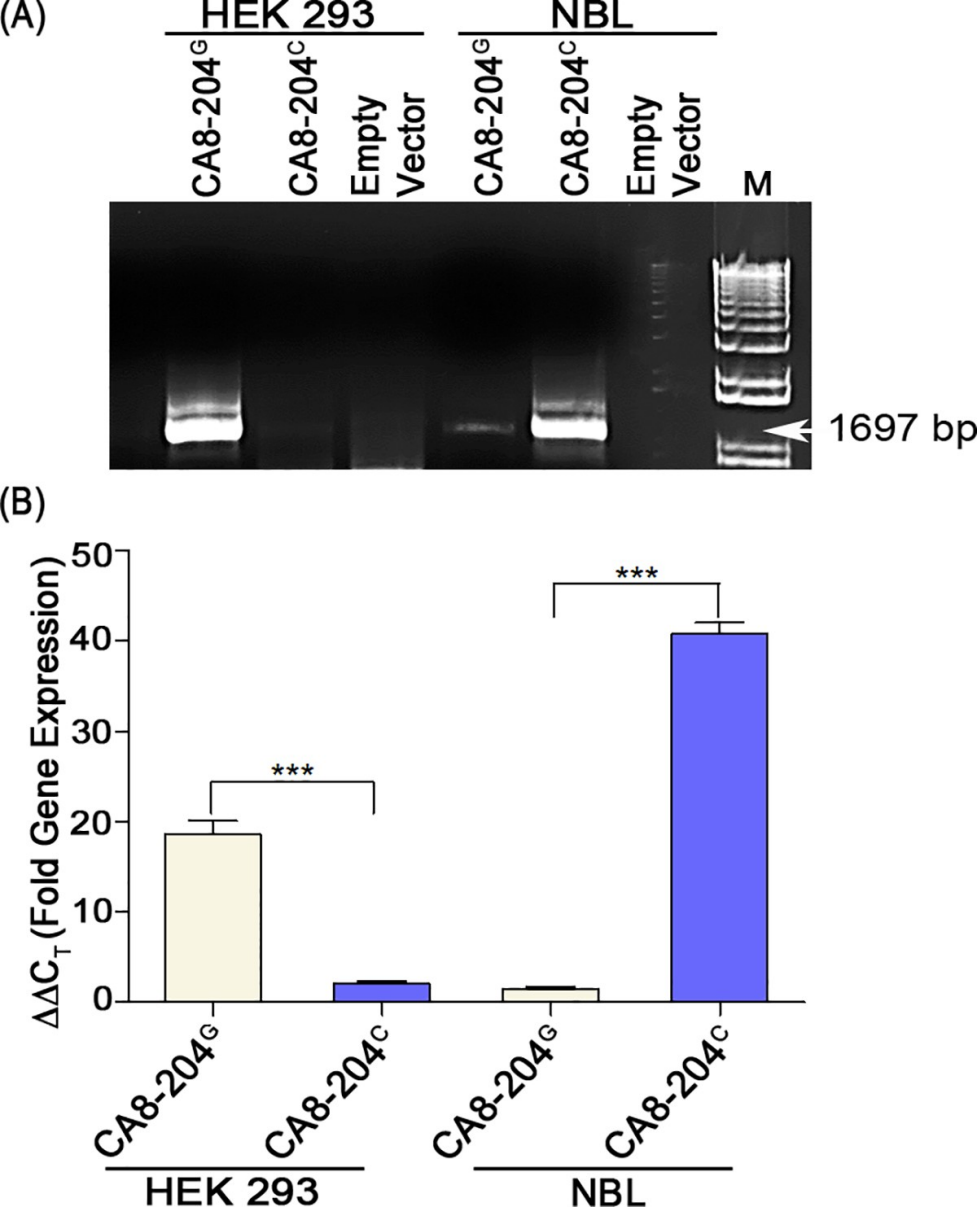
Differential tissue expression of CA8-204 variants *in vitro*. (**A)** cDNA expression from RNA (reverse transcriptase-PCR, Promega) in HEK293 (left) and NBL (right) cells transfected with empty vector, CA8-204^G^ or CA8-204^C^ using primers specific for full-length CA8-204 variants, flanking the 3'UTR. (**B)** Real-time qPCR (SYBR-green Taq, Applied Biosystems) was carried out using primers specific for the 3’ UTR region of CA8-204 in HEK293 and NBL transfected with vectors carrying either CA8-204^G^ or CA8-204^C^ or CA8-204^.^ Quantitation was done using one–way ANOVA followed by Tukey’s post-hoc test between the two groups, data was normalized by β-actin, N = 4, ***P<0.001 (GraphPad Prism software).

To better understand how rs6471859 regulates alternative splicing of CA8-204 we developed two constructs (CA8-204^G^ or CA8-204^C^) and transfected HEK293 (renal-derived, non-neuronal) and NBL (neuroblastoma, neuronal-derived) cell lines with pCMV-N-FLAG-tagged vectors coding for the naturally occurring CA8-204^G^ transcript or CA8-204^C^ produced by reversion mutation of CA8-204. We then ran qPCR (4 replicates from at least 3 separate cultures) to quantify the production of these transcripts. QPCR of the cDNA from CA8-204^G^ and CA8-204^C^ showed selective expression in HEK293 and NBL cells, respectively (**Fig 2**). Results from qPCR revealed enriched or unique expression of CA8-204^G^ in non-neuronal cells (HEK293) compared with low levels of exogenous expression of CA8-204^C^. The reverse results occurred in NBL cells, where CA8-204^C^ expression (reversion mutation as a control to test for the lack of alternatively spliced transcript) was greatly enriched over CA8-204^G^ expression (**Fig 2**).

### CA8-204^G^ produces a stable truncated peptide in a tissue-specific manner

**Fig 3:**
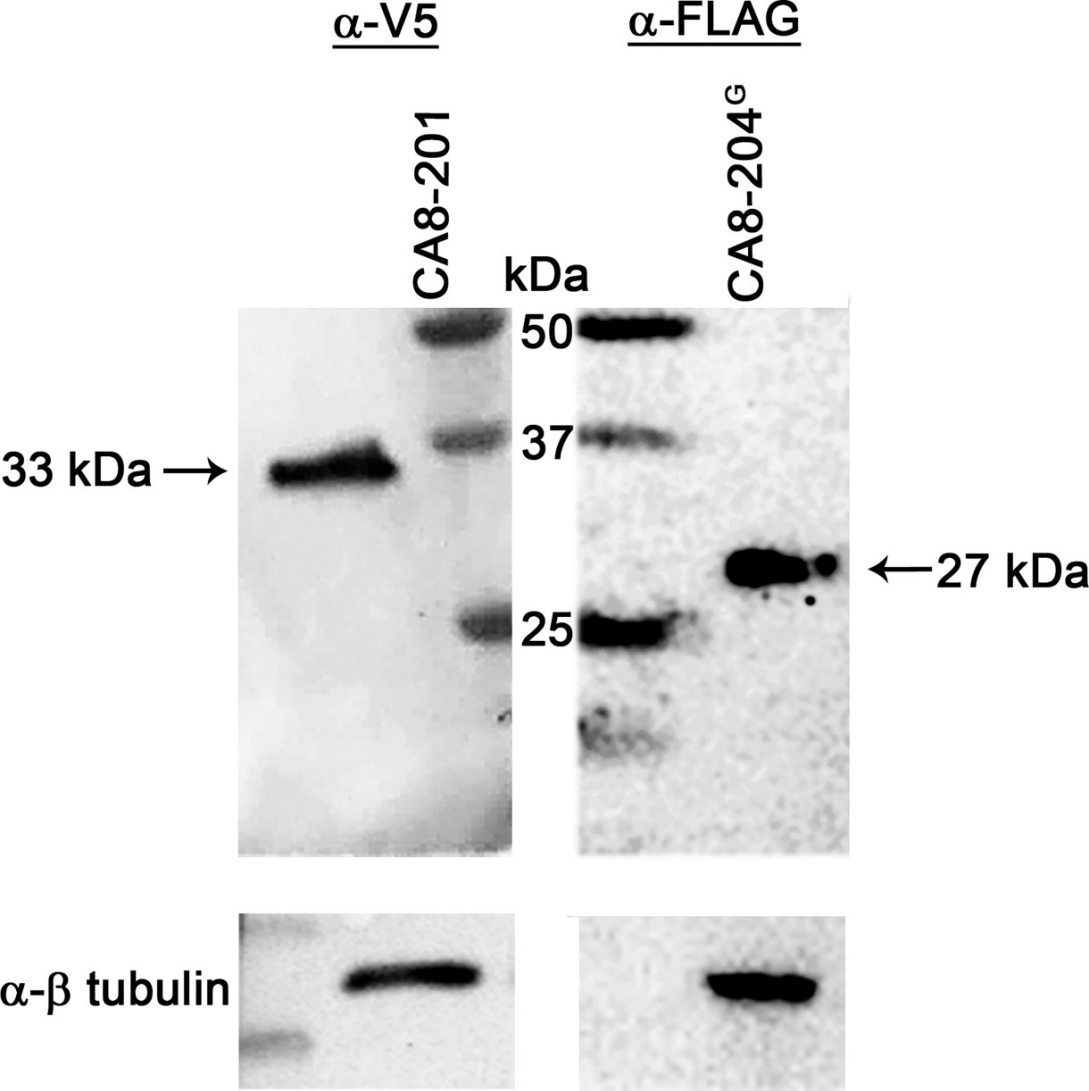
Overexpression of CA8-204 produces truncated protein product. Proteins were overexpressed through transfection with V5-CA8-201 and FLAG-CA8-204^G^ in HEK293 cells and Immunoblotted against anti-V5, anti-FLAG (Sigma Aldrich) and anti-β tubulin (control) (Cell Signalling) antibodies, bands normalized with β-tubulin. The immunoblots on left panel represent V5-CA8-201, The right panel represent FLAG-CA8-204^G^, size comparisons are facilitated by markers (middle). The truncated product from CA8-204^G^ (~27 kDa) distinguished in size from the WT CA8-201. We were unable to find expression of CA8-204^G^ in NBL cells.

To determine whether CA8-204 produced a stable peptide, we transfected HEK293 cells with CA8-204^G^, CA8-201 or empty vector and collected cell lysates for immunoblotting with anti-FLAG or anti-V5 antibodies, normalizing these data to ß–tubulin. Results are shown in (**Fig 3**) suggested that like CA8-201, CA8-204^G^ produced a peptide of the expected size of 153 amino acids (26-27kDa). However, this protein was not detected using similar immunoblotting methods with NBL cell lysates. IHC analyses *in vitro* using HEK293 and NBL cells, shows no detectable CA8-204^G^ found in NBL cells **(S1 Fig).**

### CA8-204^G^ peptide inhibits pITPR1 and ATP-induced calcium release in a tissue-specific manner

**Fig 4:**
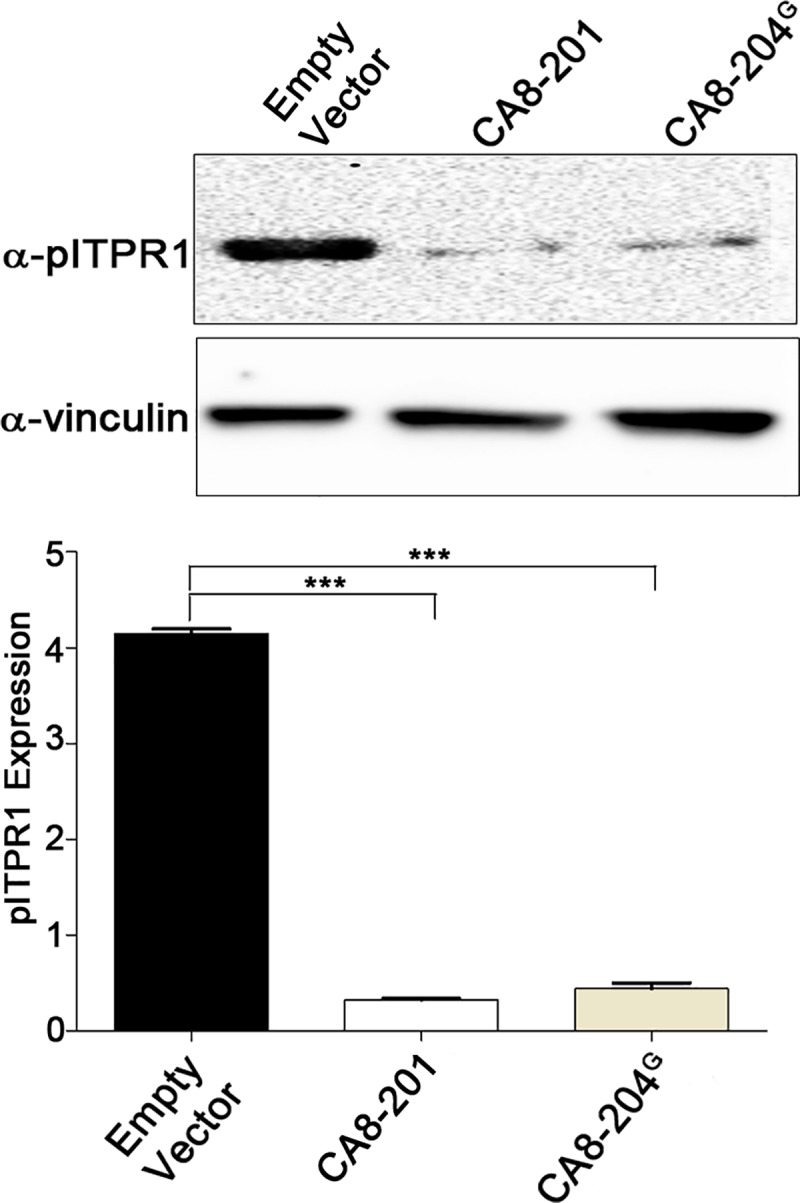
CA8-204 inhibits the phosphorylated dependent activation of ITPR1 in HEK293 cells. Immunoblot of pITPR1 (forskolin-induced phosphorylation; 10μM forskolin) in HEK293 cells, transfected with empty vector, CA8-201 (WT) or CA8-204^G^. Immunoblots suggest inhibition of pITPR1 expression through CA8-204^G^ overexpression, similar to CA8-201. Quantitation of pITPR1 expression was performed using ImageJ software. Data were normalized with vinculin. N = 3, ***P<0.001, **P<0.01. Quantitative analysis was performed using the one-way ANOVA followed by Bonferroni post–hoc test for each possible comparison (GraphPad software).

We then investigated the role of CA8-204^G^ in the regulation of ITPR1 activation (pITPR1) by measuring forskolin-induced phosphorylation in transfected HEK293 cells followed by western blotting. Our results demonstrate CA8-204^G^, similar to CA8-201, inhibits the forskolin-induced phosphorylation *in vitro* (**Fig 4).** In contrast, CA8-201, but not CA8-204^G^ inhibited phosphorylation in forskolin-induced NBL cells (**S2 Fig**).

**Fig 5:**
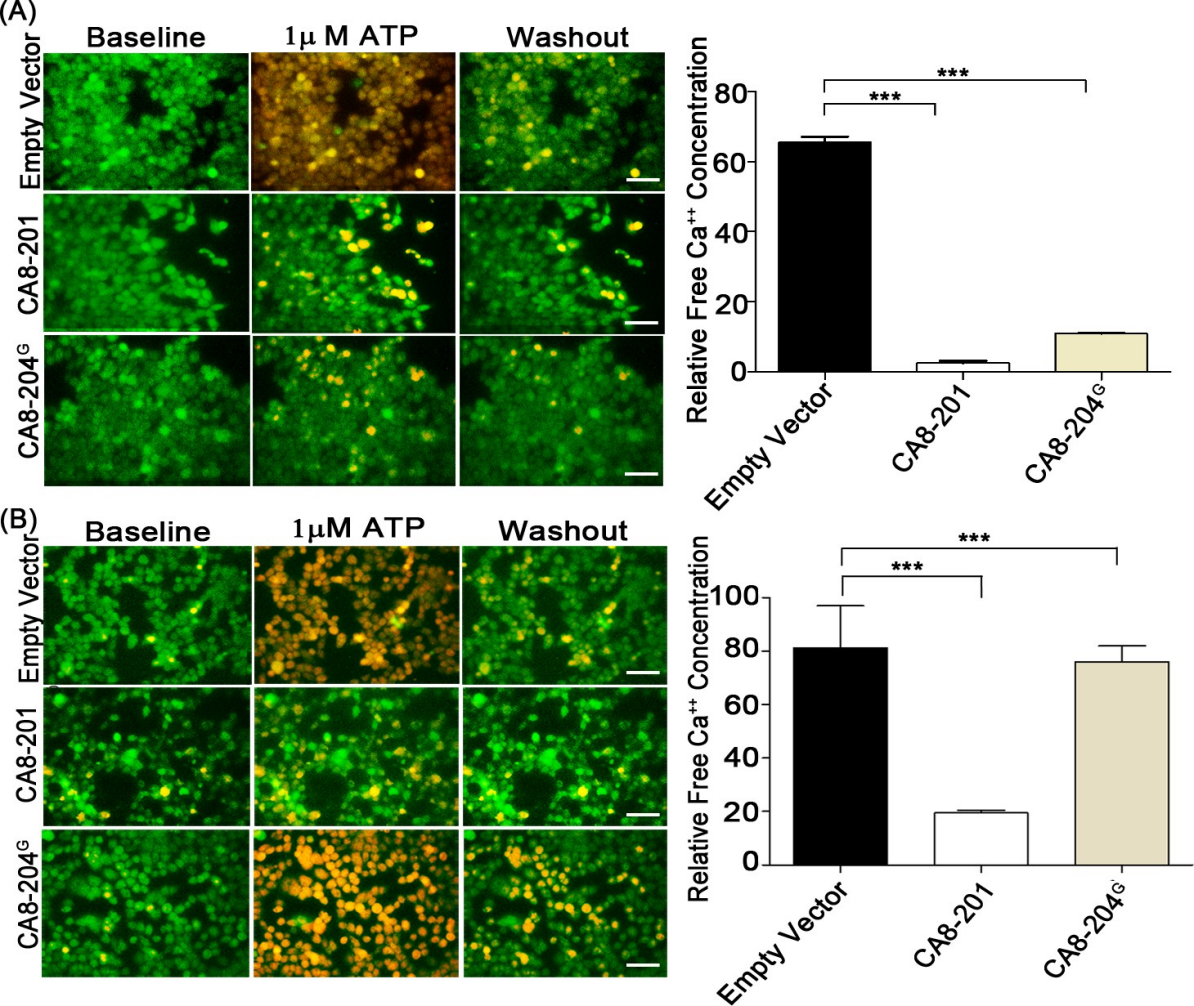
CA8-204^G^ inhibits calcium release in HEK293. **(A)** ATP dependent free calcium release (monitored by Fura-2) in HEK293 cells transfected with empty vector (negative control), CA8-201 and CA8-204^G^ are shown in images. Results from calcium imaging suggest the regulation of calcium release by CA8-201 and CA8-204^G^ in HEK293 cells, through inhibition of intracellular calcium release. Quantitation for intracellular calcium concentrations in HEK293 was performed using one-way ANOVA, post-hoc test comparisons between three groups were carried out by Bonferroni’s comparison (Graphpad Prism software) N = 6, done in triplicates, ***P <0.001, **P<0.01, scale = 40μm. **(B)** ATP dependent free calcium release (monitored by Fura-2) in NBL cells transfected with empty vector, CA8-201 and CA8-204^G^ shown in images (left). Results from NBL cells suggest negative regulation of calcium release by CA8-201, through inhibition of ITPR1. Quantitation for intracellular calcium concentrations in NBL cells was performed using one-way ANOVA, post-hoc test comparisons between three groups were carried out by Bonferroni’s comparison (GraphPad Prism software) N = 6, done in triplicates, ***P <0.001, **P<0.01.

We next investigated whether CA8-204^G^ affects ATP-stimulated ITPR1-dependent cytosolic calcium release. Once again, HEK293 and NBL cells were harvested after transfection with CA8-204^G^, CA8-201 or empty vector. ATP (1μM) was used to induce free calcium release *in vitro*, and cells were monitored in real time by measuring calcium release (Fura2-AM dye). Empty vector had no effect on ATP-mediated free calcium release. HEK293 cells transfected with either CA8-201 or CA8-204^G^ demonstrated reduced cytoplasmic free calcium after ATP-stimulation **(Fig 5A)**. In contrast, only CA8-201 inhibited ATP-stimulated calcium release in NBL cells (**Fig 5B**). Collectively, these results demonstrate tissue-specific transcription, translation and functions of CA8-204^G^ on calcium signaling *in vitro* is restricted to non-neuronal cells.

### Overexpression of CA8-204 inhibits thermal hypersensitivity after inflammatory pain in mice

**Fig 6:**
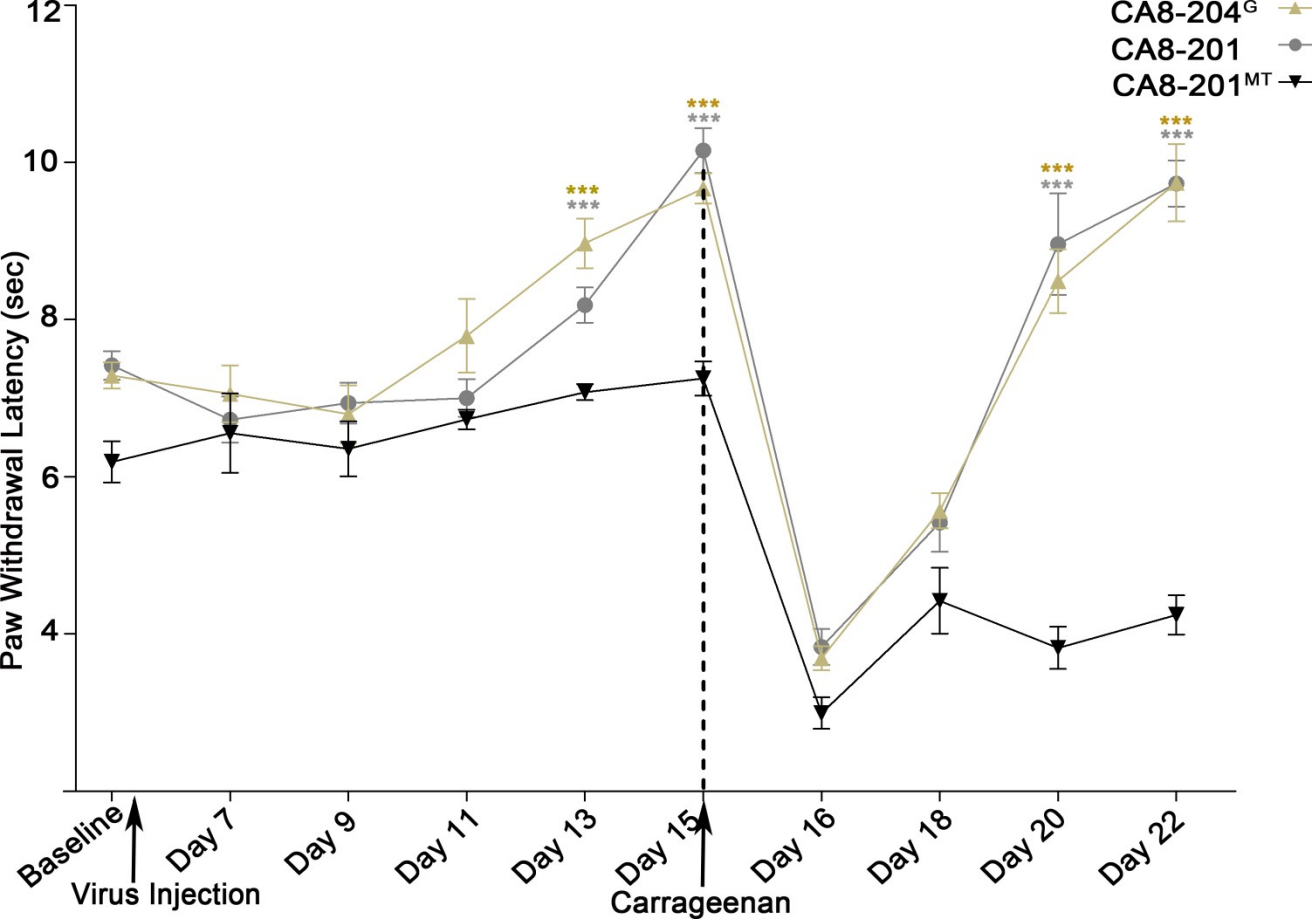
CA8-204^G^ produces analgesic and anti-hyperalgesia similar to CA8-201 in carrageenan inflammatory pain models. Gene transfer of AAV8-FLAG-CA8-204^G^ (CA8-204^G^); AAV8-V5-CA8-201^WT^ (CA8-201^WT^); AAV8-V5-CA8-201^MT^ (CA8-201^MT^); or empty vector were injected via the sciatic nerve injections before carrageenan injection to produce an inflammatory pain model. Paw withdrawal thermal latencies were measured daily after baseline, after virus injection and/o and after carrageenan injection (left paw). Mice that received AAV8-FLAG-CA8-204^G^ (1.5μl, 1E13 genome copies/ml) after day 7 had a significantly higher threshold for pain compared to CA8-201^MT^, especially on days 13 and 15. Increasing paw withdrawal latencies reached a maximum of 10–12 seconds on day 14–15, similar to CA8-201^WT^. CA8-201^MT^ produced no similar increase in withdrawal latencies. After carrageenan injections on Day 15, all groups showed reduced paw withdrawal latencies through day 18. However, mice in the CA8-201^WT^ and CA8-204^G^ groups showed a significantly enhanced paw withdrawal latency on days 20 and 22 compared to the CA8-201^MT^, indicating the anti-hyperalgesia protection was provided by the CA8-204^G^. On days 13, 15, 20, and 22 mark the differences in paw withdrawal latency significantly greater than baseline, in CA8-201 and CA8-204^G^ compared with CA8-201^MT^. Mice in the negative control group showed paw withdrawal latency either at baseline or below baseline on these days. N = 8, ***P<0.001, **P<0.001 Two-way (repeated measures ANOVA) followed by Bonferroni’s post-hoc test statistical group test (GraphPad Prism).

Due to the tissue-specific transcription, translation and functions of CA8-204^G^ in non-neuronal cells, we investigated the impact of this alternative transcript using a pain model *in vivo*. AAV-mediated gene transfer to DRG cells including glial and sensory neurons through SN injections is an effective route in the treatment of chronic pain [5, 6]. We chose AAV8 in this study because it has been shown to transduce both neuronal and glial DRG cells after peripheral nerve injection. SN in wildtype C57BL/6J mice (N = 8, each group) was carried out using AAV8-V5-CA8-201 (positive control), AAV8-V5-CA8-201^MT^ (negative control) and AAV8-FLAG-CA8-204^G^. Thermal behaviors were monitored daily and maximum latencies were recorded through day 15 for mice receiving SN injections. Mice receiving CA8-201 or CA8-204^G^, but not those injected with CA8-201^MT^, demonstrated elevations in their thermal latencies starting at Day 13 reaching a maximum by Day 15 (two-way repeated measures ANOVA test, post-hoc test, overall P-value <0.001; CA8-201 vs. CA8-201^MT^, P-value<0.001; CA8-201^MT^ vs. CA8-204^G,^ P-value<0.001) of approximately 3.0 seconds above baseline. This increase in thermal withdrawal latencies in C57BL/6J mice exceeds a dose of 80 mg of oral morphine in a 60 kg adult, after allometric conversion [7]. Following carrageenan injection thermal paw withdrawal latencies reached a minimum on Day 16. Mice injected previously with CA8-201, CA8-204^G^, but not CA8-201^MT^ showed rapid recovery through Day 22 (**Fig 6)** (P-values<0.001) and once again demonstrating profound analgesia with significant increases over baseline in thermal withdrawal latencies after Day 20 (CA8-201^MT^ vs. CA8-201, P-value<0.001; CA8-201^MT^ vs. CA8-204^G,^ P-value<0.001).

### Distribution of CA8-204 in DRG cell populations explains profound analgesia and anti-hyperalgesia *in vivo*

**Fig 7:**
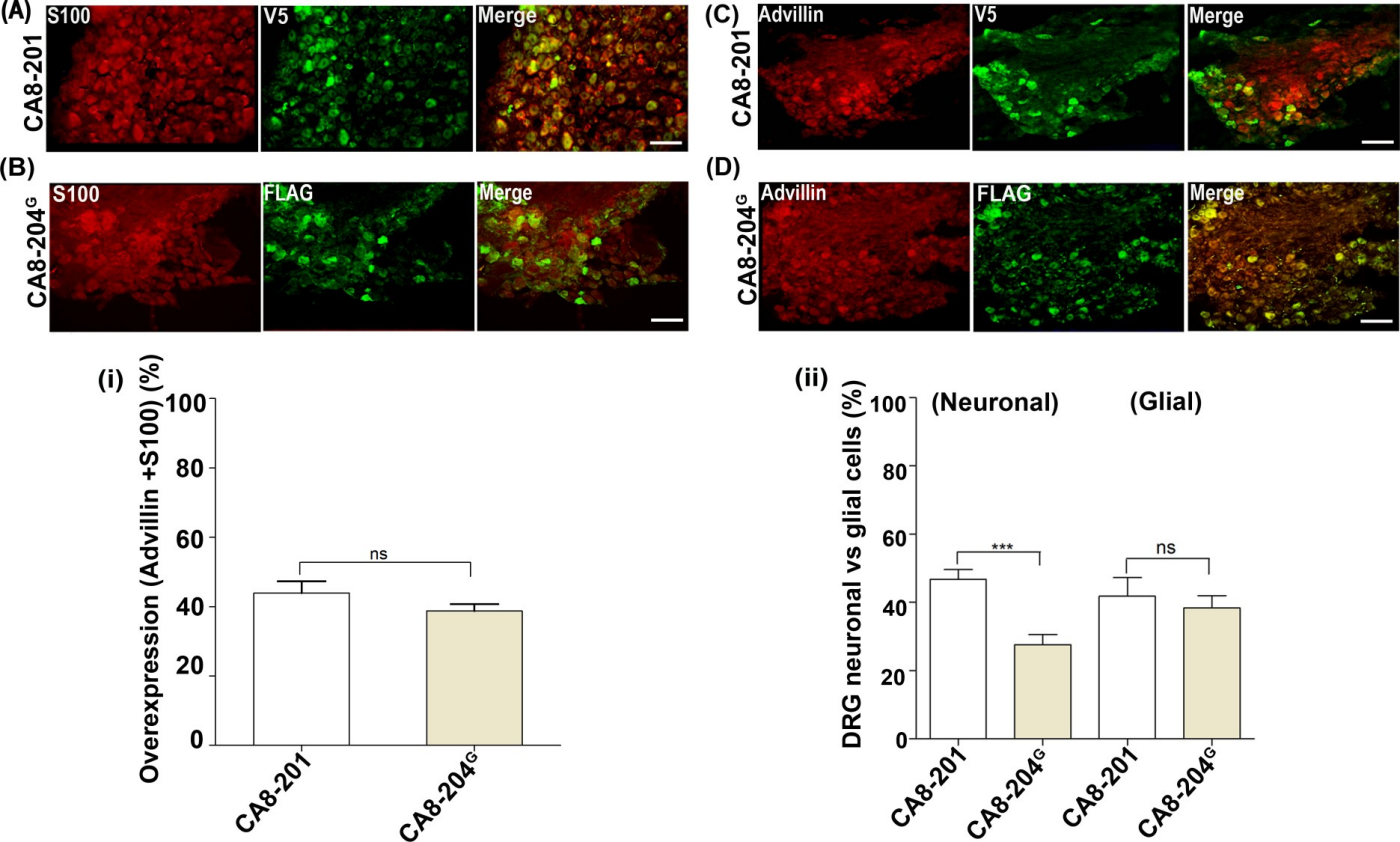
CA8-204^G^ is expressed in DRG neuroglia and neuronal cell population. Immunostaining was performed in DRG sections in mice receiving SN of AAV8-V5-CA8-201^MT^, AAV8-V5-CA8-201 and AAV8-FLAG-CA8-204^G^. **(A)** Immunostaining was done using antibodies anti-V5 for CA8-201 (V5, green) and anti-S100 (glial marker) (S100, red), and anti-V5 with anti-S100 antibodies, respectively from the merged image (Merge). **(B)** Immunostaining was performed using antibodies anti-S100 (S100, red), anti-FLAG for CA8-204^G^ (FLAG, green) and anti-FLAG and anti-S100 from the merged figures (merge); (**C)** Immunostaining using antibodies anti-Advillin (Advillin, red), anti-V5 (V5-CA8-201, green) and anti-Advillin and anti-V5 (Merge) from the merged figures was carried out in DRG sections; (**D)** Using antibodies anti-Advillin (Advillin, red), anti-FLAG for CA8-204^G^ (FLAG-CA8-204^G^, green) and anti-FLAG and anti-Advillin from the merged figures (merge). **(i)** Histogram of the IF results demonstrates CA8-201 and CA8-204^G^ co-localize with DRG neuroglia and neuronal populations. The % overexpression was calculated from the positive cells overlapping with advillin and S100 markers, divided by DAPI as counter-stain which was used. **(ii)** Overlap (%) between CA8-204^G^ and CA8-201 were calculated separately from the total population of DRG glial and neuronal cells. IF was done using DRG tissues retrieved from animals after 4 weeks after SN injection. Percentage of overexpression of CA8-201 and CA8-204^G^ and overlap in total DRG cells were quantified using one-way ANOVA with Bonferroni's post-hoc test (***P<0.001, **P<0.01, *P<0.1, ns = non-significant, N = 8 each gene group). (IF = Immunofluorescence) (Scale = 50μm).

Lumbar DRG sections (L4 and L5) were collected from each group (N = 8) after perfusion and immunohistochemistry (IHC) was performed using primary anti-FLAG and -V5 antibodies, with fluorescent (Alexa Fluor 488; green) secondary antibody. Because DRG are comprised of both neurons and glial cells, to measure the overexpression of CA8 peptides after SN, we used anti-S100 (glia) and anti-advillin (sensory neurons) antibodies with fluorescent secondary antibody (Alexa Fluor 594; red) to define the tissue-specific expression of each CA8 peptide. Total cells were counted using DAPI staining. The percentage of overexpression in neuronal and glial cells was measured separately for V5-CA8-201^MT^, V5-CA8-201 and FLAG-CA8-204^G^ **(Fig 7A, 7B, 7C and 7D, S3 Fig**). While overexpression calculated for total population of cells (neurons and glia) in CA8-201 and CA8-204^G^ showed a very similar pattern (**Fig 7(i)**), surprisingly, and in contrast to our *in vitro* data, significant CA8-204^G^, like CA8-201, was present in both neuronal and glial cells. However, while CA8-204^G^ expression was not significantly different from CA8-201 in glial cells, expression was reduced (about 50%) when compared with CA8-201 in neuronal cells (**Fig 7(ii)**). Nonetheless, this level of neuronal expression *in vivo* appears to be enough to produce analgesia and anti-hyperalgesia in this animal model. Thus, moderate levels of the functional CA8-204 peptide appear to be protective *in vivo*. Thus, the “all” or “none” expression based on cell type *in vitro* was not replicated *in vivo*. Presumably, even this moderate level of expression of CA8-204 in neuronal cells explains how G/G homozygotes evade a deleterious phenotype.

### Alternative splicing of CA8-204 is associated with creation of a cryptic splice site at rs6471859

We performed a bioinformatic analysis using Human Splicing Factor (HSF 3.1) web server to understand the role of rs6471859 as a *cis*-acting regulatory element within the intronic or exonic sequences of CA8-204 [9]. Human Splicing finder or HSF3.1 (http://www.umd.be/HSF3/HSF.shtml) predicts splice sites (donor or acceptor) by calculating maximum entropy for the target nucleotide sequence. Consensus values range from 0 to 100 for HSF (Human Splicing Factor). The threshold is defined at 65 for HSF, which means that every signal with a score above this threshold is considered to be a splice site (donor or acceptor). The webserver also predicts the nature of the splice site.

The program was run for sequence CA8-204^G^ including the naturally occurring “G” allele at position 1,416 in the 3’UTR; and on CA8-204^C^ (reversion mutation) by replacing the “G” with a “C” nucleotide at rs6471859. **Table 2** provides results of HSF3.1 analyses. The “G” allele at rs6471859 is predicted to create a donor site within the sequence “CA**G**GTCTTG” with CAG as potential new splice site (71.92% consensus). This splice site is lost with the “C” allele (CA**C**GTCTTG) at rs6471859. These findings suggest a potential mechanism associated with the observed alternative splicing of the CA8 gene.

**Table 2:** Splicing analysis in CA8-204 reveals cryptic splice sites in rs6471859 created with “G” allele. Results of splicing analyses from HSF3.1 (http://www.umd.be/HSF3/HSF.shtml) for the CA8-204 sequence (NCBI accession: NM_001321837.1, NG_023193.2, refSNP or clinical variation: rs6471859) with the “G” allele at 1,416 bp (SNP location within CA8-204 transcript) and when it is replaced with “C” allele at position 1,416 bp (SNP variation) 0–100, < -10: splice site disrupted; >30: splice site formed. Consensus value is calculated based on the possibility of production of a cryptic splice site within the sequences, ranging from 0–100, <10- splice site disrupted; >30- Splice site formed. The sequence containing the SNP has been highlighted in bold and underlined.

Splice Site Type	rs6471859 (“G” vs C” allele)	New potential splice site	Consensus Value (0–100)
Donor	CA**G**GTCTTG	**CAGgtcttg**	**71.92**
None	CA**C**GTCTTG	None	-

## Discussion

In this report we identify the following for the first time: (1) a human DRG exon-level *cis*-eQTL (rs6471859) that regulates alternative splicing of CA8; (2) a cryptic splice site is produced by the “G” allele at position 1,416 (rs6471859) within the 3’UTR of the CA8-204 transcript; (3) CA8-204^G^ (naturally occurring transcript) and CA8-204^C^ (reversion mutation) expression constructs demonstrate tissue-specific splicing *in vitro*; where CA8-204^G^ is transcribed predominantly in non-neuronal cells (HEK293), CA8-204^C^ is transcribed predominantly in neuronal-derived cells (NBL); (4) Surprisingly, we also show that CA8-204^G^ is translated into a stable truncated peptide in HEK293 cells; (5) and in contrast to the work of Hirota *et al*., (2003), CA8-204 inhibits forskolin-induced ITPR1 activation (pITPR1) and ATP-stimulated calcium release [3]. Collectively, our *in vitro* data demonstrate tissue-specific transcription; translation and functions of CA8-204^G^ are restricted predominantly to non-neuronal cells. Therefore, it appears that homozygous G/G individuals exclusively produce CA8-204, predominantly in non-neuronal DRG cells. These results raise numerous important questions because according to 1000 Genomes Project Phase 3 allele frequencies for rs6471859; G/G homozygotes are common across in almost all populations. To further address the presumed discordance between these *in vitro* results with what is known about the deleterious effects of CA8 deficiency, we show: (6) AAV8-mediated gene transfer of CA8-204 via direct sciatic nerve injection to transduce DRG *in vivo* showing CA8-204 overexpression of CA8-204, like CA8-201, produces profound analgesia after about 13 days; (7) overexpression of CA8-201 and CA8-204^G^ showed very similar patterns in DRG neuronal and glial cells **(Fig 7 (i));** and (8) surprisingly, while CA8-204^G^ expression was seen in neuronal cells, but to a lesser extent (about 50% compared to CA8-201)(**Fig 7(ii)**). Importantly, this level of neuronal expression *in vivo* appears to be enough to produce anti-hyperalgesia and profound analgesia in the inflammatory pain model tested. Thus, it is critical to appreciate that despite its moderate levels, the functional CA8-204 peptide in somatosensory cells appears to be protective *in vivo*. This is consistent with the described CA8-S100P null mutation that only produces a phenotype in recessive homozygotes [4].

GTEX data reported for rs6471859 show it represents a multi-tissue eQTL including both neuronal and non-neuronal tissues as follows: lung (4.0e-23); whole blood (8.8e-6); brain-hippocampus (2.7e-4), -cortex (3.5e-3), -cerebellum (2.3e-3), and -pituitary (3.2e-7). However, no splice eQTL data are reported (https://gtexportal.org/home/snp/rs6471859). It is also important to note that our *in vitro* data could have been misleading if we wrongly concluded that there is an “all” or “none” expression of CA8-204 based eQTL data by cell type. Only the *in vivo* pain model data presented enabled us to explain the discordance between the population genetic data for rs6471859 and the lack of a severe phenotype in G/G homozygotes due to the splice variants reported. Dorsal root ganglia (DRG) comprises both of neuronal and neuroglia population of cells. Markers such as S100 and advillin were used to identify glial and neuronal cell population. Glial cells in form of satellite glial cells and sensory ganglia surely regulate the afferent signaling and neuronal excitability, with a possible role in chronic pain formation as shown in literature [10–12]. Again, the moderate level of expression of CA8-204 in neuronal cells *in vivo* explains how G/G homozygotes evade a deleterious phenotype. Our overall findings are consistent with reports suggesting that CA8 null mutations producing severe spinal cerebellar ataxia are recessive, and heterozygotes are phenotypically normal. Thus, heterozygotes producing approximately 50% of normal cellular CA8 evade a severe phenotype [4]. Surprisingly, CA8-204 transcript is stably expressed in a tissue-specific manner *in vitro*. This may likely occur because of this retained intron harboring a polyadenylation signal. Retained introns with self-contained 3’UTR polyadenylation signals are prevalent in animals [13–15]. Several alternatively spliced gene products prove to be beneficial and provide potential therapeutic strategies in treatments of diseases such as cancer, spinal muscular atrophy and tauopathies leading to neurodegenerative diseases [16–19]. Alternative Polyadenylation (APA) generates protein isoforms with truncated/shortened protein products such as vascular epidermal growth factor regulator (VEGFR) and SMN (Spinal Motor Neuron 1 and 2) resulting from intron retention [20–22]. 3′ UTR length is also a critical factor towards regulating transcript stability and protein expression. In general, the longer the 3′ UTR, the greater the likelihood for sequence-specific motifs to be recognized by splicing and translational regulatory factors [21, 23]. Positioning of the intron adjacent to an alternatively spliced exon increases the likelihood that it is retained. Recent studies suggest that immortal cell lines have differential recruitment and highly altered RNA-splicing genes representing an underappreciated hallmark of tumorigenesis [24–26]. Differential recruitment of specific splicing factors may explain our tissue-specific splicing results *in vitro* (**Fig 2**).

Remarkably, our results demonstrated that CA8-204 also produces a stable functional peptide, which contradicts the earlier findings of Hirota *et al*. (2003), which showed that virtually the entire CA8 peptide (exons 1–8) is required for the negative regulation of IP3 binding to ITPR1 [3]. Although, we didn’t show physical binding data, it is clear from our results that CA8-204, like CA8-201 inhibits ITPR1 activation by phosphorylation and intracellular calcium release. We speculate that the difference between our findings and theirs may be attributed to the use of purified ITPR1 for the IP3-CA8 binding. It is likely that the functions of ITPR1 are highly dependent associations with Homer, TRPC1, 80 K-H, IRAG, cGKI, IRBIT, CIB1, Na^+^-K^+^-ATPase and protein 4.1N, which are shown to dynamically impact channel activation [27–34].

Our results clearly demonstrated that CA8-204 overexpression is protective of inflammatory pain *in vivo*, suggesting that this peptide is fully functional and may serve as a validated therapeutic candidate as a long acting local anesthetic using gene therapy.

## Materials and methods

### Ethics statement

Director of Compliance, IACUC/IBC/ESCRO, Office of Research at University of Miami, Miller School of Medicine approved the protocol for the use of animal subjects in research study (IBC #17–075). Before SN injections all mice were anesthetized using intraperitoneal injection of a cocktail of ketamine, xylazine and acepromazine (VEDCO, Saint Joseph, Mo). Perfusion was carried out only once mice were anesthetized with isoflurane. The tests for assessing neuropathic pain were performed using IACUC approved protocols and the thermal beam projected for 15 seconds taken as cut-off threshold to prevent potential injury.

### Human DRG eQTL analyses

CA8 exon-level DRG analyses were run essentially as described elsewhere [35]. Briefly, bilateral L4 and L5 DRG were collected from a total of 214 brain-dead human subjects following asystole after consent of family members and snap-frozen. Genomic DNA was isolated and genotyped using Illumina’s Infinium Human Omni Express Exome-8 v1.2 chip (≈ 1M probes) and analyzed using Illumina Genome Studio 2011.1, as described. RNA was isolated from the same DRG samples frozen in TRIzol reagent (Qiagen, Austin, TX). Total RNA was analyzed using Affymetrix Human Transcriptome Array 2.0 (≈ 70K gene-level probes, ≈ 900K isoform-level probes). All statistical tests were performed with age, gender, sample’s average expression and the first two eigenvectors (from principal components analysis to capture racial/ethnic differences) per chromosome as covariates. eQTL discovery was performed on CA8 utilizing 20 exon-level probes that recognize 4 unique CA8 gene transcripts with an expression intensity level above 3. Dataset for eQTL results can be found at GSE78150 (Geo Accession; NCBI).

### Generation of V5 tagged CA8 wild type, mutants, pCMV-N-FLAG and AAV-ITR vectors expressing CA8-204 alternative variants

An expression construct containing the naturally occurring CA8-204 transcript (NCBI accession: NM_001321837.1) including the G allele at nucleotide position 1416 (CA8-204^G^) was generated from in pCMV-Sport6 vector (Harvard Facility) and cloned using PCR with forward primer 5’-GGGGACAACTTTGTACAAAAAAGTTGGCATG GCGGACCTGAGCTTC-3’ and the reverse primer: 5’-GGGGACAACTTTGTACAAAAAAGTTGGCATGGCGGACCTGAGCTTC-3’ and cloned into pCMV-FLAG(DYKDDDDK)-N-terminal vector (Takara), with forward primer 5’-TTTGTCGACAACGCACGCCTGCTTGCAC-3’ and reverse primer 5’-TTTTGGTACCTTACAGTAATGCTGTCAAACACTTCAACAG-3’. The restriction enzymes SalI and KpnI (NEB) were used for restriction digestion on PCR products and for the pCMV-N-FLAG (Takara) vector having similar restriction sites. CA8-204^C^ was produced using GENEART site-directed mutagenesis system (Invitrogen Life Technologies, Carlsbad, CA) and specific primers (forward: 5’-GTTGGATTCAGTCCACGTCTTGATGTTATTT-3’, reverse: 5’-AAATAACATCAAGACGTGGACTGAATCCAAC-3’) were employed to create one nucleotide substitution at position 1416 in the CA8-204 transcript. This substitution was in the CDS sequence using the forward primer: 5’-ATGCAGATAGAAGAATTTCGACACATGTCAAGGGGGCAGA-3’ and the reverse primer: 5’-TCTGCCCCCTTGACATGTGTCGAAATTCTTCTATCTGCAT-3’. The pCMV-N-FLAG constructs containing CA8-204^G^ and CA8-204^C^ sequence was confirmed using Sanger sequencing. Human wildtype CA8 cDNA (CA8-201) was purchased from Origene (NCBI accession: NM_004056). The plasmid was digested with enzymes BamH1 and Not1 (NEB). The purified cDNA was ligated with linearized pAAV-MCS vector using quickstep ligation (Invitrogen) and transformed. The sequence of the insert was confirmed through sequencing. The CA8-201 cDNA was mutagenized to produce the S100P mutation (CA8-201^MT^), as described previously [5].The transfer of CA8-204 to AAV-ITR vector through the PCR amplification of pCMV-N-FLAG–CA8-204^G^ (CA8-204^G^) using primers specific for 1,697 bp long alternative variant of CA8 containing FLAG tag carrying BamHI and XhoI sites; forward primer: 5’-TTTGGATCCGCCACCATGGACTACAAGGACGACGATGA-3’ and reverse primer: 5’-TTTTCTCGAGTTACAGTAATGCTGTCAAACAC-3’. PCR fragments were restriction digested with BamHI and XhoI (NEB), purified and ligated with linearized pAAV-MCS (ITR) vector between BamHI and XhoI sites. The constructs were confirmed using Sanger sequencing. The recombinant AAV8-V5-CA8-201, AAV8-V5–CA8-201^MT^, AAV8-FLAG-CA8-204^G^ viral particles were produced by the Miami Project Viral Vector Core at the University of Miami Miller School of Medicine, essentially as described previously [5]. The purified AAV particle titers were at least 1x 10^13^ genome copies per mL.

### Cell preparations and transfections

HEK293 and NBL (neuroblastoma, ATCC) cells, passed at least three times with 0.5% trypsin in DMEM/F12 (GIBCO) complemented with penicillin/streptomycin and 10% fetal bovine serum (FBS; GIBCO) were allowed to grow at the rate of 4 x 10^5^ cells for 6-well plates and 2 x 10^5^ cells for 12 well plates, for obtaining a 80–90% confluent layer after 24 hours. Briefly, all transfections were performed using LTX with plus reagent or Lipofectamine 2000 according to the manufacturer’s instructions (Invitrogen). All transfections were performed in Opti-MEM I reduced serum medium (Invitrogen) with 2 μg DNA and 6 μl Lipofectamine 2000 for 6-well plates or 0.5 μg-1 μg for 12-wells. Cells were maintained for 4–6 h (minimum of 2h) in the transfection media at 37^o^ C/ 5% CO_2_ followed by replacing it with DMEM/F-12 (1:1) plus FBS and penicillin-streptomycin. A 48 h incubation of cells in these conditions is sufficient for their use in measurements of mRNA and protein expression using RNA extraction followed by RT (reverse transcriptase)- PCR and/or real-time PCR using SYBR-green PCR master mix on the Step One Plus real time PCR machine (Applied Biosystems), western blot and immunoprecipitation studies.

### Cell culture RNA extraction, reverse transcription-polymerase chain reaction (RT-PCR) and real time PCR (qPCR)

Total RNA was extracted from cultured HEK293 and NBL cells transfected with CA8-204^G^, CA8- 204^C^, CA8-201 and CA8-201^MT^ expression constructs using the RNeasy RNA extraction kit (Qiagen) following the manufacturer’s protocol. Total RNA was quantified by an Epoch spectrophotometer (BIOTEK). Two-step RT-PCR was performed using the Access-quick RT-PCR system (Promega) and Faststart DNA Polymerase (Roche) according to the supplier’s protocols. RT-PCR was used to amplify exogenous CA8-204^G^ and CA8-204^C^ with the forward primer: 5’-ATGGACTACAAGGACGACGATG-3’ and the reverse primer: 5’-GGGCTATTTTCTGGGGTAAA-3’. For the RT-PCR, the PCR products were loaded on 1.2% agarose gel, and were visualized with ethidium bromide. No template was used as a negative control. Quantitative PCR was used to amplify exogenous CA8-204^G^ and CA8-204^C^, flanking the 3’UTR region, with the forward primer: 5’-ATGGC GCTGGCCCCATGGGTT-3’ and the reverse primer: 5’-GGGC TATTTTCTGGGGTAAA-3’. ACTB (ß-actin) was used as internal control with primers forward: 5’-AAATCTGGCACCACACCTTC-3’ and reverse: 5’-CACCTTCACCGTTCCAGTTT-3’ (Sigma Aldrich, St. Louis, MO). qPCR and analysis was carried out on a Step One Plus system (Applied Biosystems, Invitrogen) using Power SYBR-green PCR master mix (Applied Biosystems). Primers (final concentration taken as 250nM) were designed across the beginning and end of the 3’UTR of the CA8-204 alternative variants with retained intron. No endogenous CA8-204^G^ or CA8-204^C^ were detected at baseline in either cell line. Quantitation of CA8-204^G^ or CA8-204^C^ was normalized to the expression of ß-actin (ACTB). Primer efficiency was determined using the melting curve analyses after qPCR.

### Western blotting

Transfected and non-transfected HEK293 and NBL cell cultures were lysed in RIPA buffer complementing with appropriate concentrations (1μM) of proteinase and phosphatase inhibitors (Sigma). Cell lysates containing proteins were separated on 4–15% or 10% SDS polyacrylamide gels and transferred to a PVDF membrane (Biorad) using transfer buffer containing 20% methanol in 25mM Tris-HCl and 192mM glycine. For transfer of large proteins such as pITPR1 western blot, 5–6% Tris-HCl/SDS polyacrylamide gel and 10% methanol in transfer buffer were used to increase the transfer efficiency. Blocking of the membrane was done with 5% skimmed milk in Tris-buffered saline (TBS) with 0.1% tween-20 for 1 h at room temperature followed by wash with TBS containing tween-20 and incubation with primary antibodies at 4°C. The blots were then incubated for 1 h at HRP-conjugated secondary antibodies at room temperature (Santa Cruz laboratories). Pierce Super Signal substrate (Thermofisher Scientific, Rockford, IL), was used to visualize bands. Primary antibodies used were as follows: anti-CA8 (Santa Cruz, Santa Cruz, CA), anti-V5 (Invitrogen), anti-pITPR1 (Cell Signalling Tech.) and anti-ITPR1 (Cell Signaling Technology, Ser-1755), anti-FLAG (Sigma Aldrich), anti-FLAG (Aves), anti-Vinculin (Abcam) and anti-β-actin (Cell Signaling Tech.).

### Calcium imaging

HEK293 and NBL (ATCC) cell lines, were passaged at least three times and were transfected with vectors vehicle (empty vector with FLAG tag), CA8-201 and CA8-204^G^ 24 hours after the seeding cells on the 12 well plates. Cells (1x 10^5^) were split onto 12 mm glass coverslips (Propper, Long Island, NY), previously coated with poly-lysine (12h) and laminin for 2 h. Transfections were done as described above. Cell media was replaced with Fura-2 dye from the Fluo-4 Calcium Assay Kit (Life Technologies) on the day of the assay followed by the incubation at 37°C for 30 minutes. Cells were moved to the room temperature, washed briefly twice with calcium containing buffer (buffer I; concentrations in mM, 130NaCl; 4.7 KCl; 2.3 MgSO_4_; 5 Glucose; 20 HEPES; 1.2 KH_2_PO_4_, Calcium Chloride; pH 7.4). Coverslips were loaded in the upright position on the microscope washed and perfused with Ca^2+^-free media containing EGTA (concentrations in mM: 130 NaCl; 4.7 KCl; 2.3 MgSO_4_; 5 Glucose; 20 HEPES; 10 EGTA; 1.2 KH_2_PO_4_, pH 7.4). Each coverslip was allow to equilibration for 5 minutes before imaging. Cells were visualized at every 2 seconds for 10 minutes while Ca^2+^ free buffer alone or media containing ATP were perfused onto the coverslips with cells. Individual cells were analyzed using the LAX system on Leica microscope (Leica Microsystems Inc, Buffalo, IL).

### Preparation and care of animals

All experiments and procedures performed on animals were conducted in compliance with the guidelines from the Institutional Animal Care and Use Committee (IACUC) at University of Miami for the care and use of laboratory animals and the current guidelines for experimental pain in conscious animals, following an IACUC approved protocol [36, 37]. Male adult C57BL/6J mice, 8–12 weeks of age and weighing 25–35 g, were acquired from Jackson Laboratories (Bar Harbor, Maine, USA). Total of 5 mice were kept in home cage environment with access to food and water *ad libitum*. All animals were allowed to acclimatize for at least 10 days and were housed in a 12–12 h light–dark cycle in a sterile facility under controlled humidity and temperature.

### Sciatic nerve injection of AAV8 viral particles for gene transfer *in vivo*

Viral particles from AAV8-V5-CA8-201 (CA8-201) were used in this study as positive control group. As previously shown, analgesic and anti-hyperalgesic activity is abolished in the (S100P) null mutant AAV8-V5-CA8-201^MT^ (CA8-201^MT^) [6], that was used as a negative control; and AAV8-FLAG-CA8-204^G^ (CA8-204^G^) was used as the test group. SN (sciatic nerve) injections of viral particles was followed using a previously prescribed procedure [7]. We have used V5 and FLAG tags due to their small size as tags, and data that have shown these tags lack effects in neurobehavioral studies [5, 6, 38–40]. Preceding the SN exposure, 1.5 μl of 1.0E13 genome copies/ml of viral particles were injected into the sciatic nerve through a 35-gauge NanoFil needle using a NanoFil syringe (World Precision Instruments, Sarasota, FL). While the injection in all groups, the mice were anesthetized using intraperitoneal injection of a cocktail of ketamine, xylazine and acepromazine (VEDCO, Saint Joseph, Mo). The sciatic nerve injection was performed at a distance of 45 mm from the third toe. Needles were slowly removed at approximately 1 minute after sciatic nerve injection. A number of studies show that chronic and neuropathic pain is regulated by the sex differences [41, 42]. We did not observe significant sex-specific differences in CA8-204 transcript expression in our small DRG sample. In addition, GTEX data show no sex-specific differences in gene expression for CA8 across most tissues (GTEx Analysis Release V7 (dbGaP Accession phs000424.v7.p2; https://gtexportal.org/home/snp/rs6471859). Therefore, to minimize the number of animals used in these studies, we selected male C57BL/6J mice only. This represents a limitation of the current study.

### Carrageenan inflammatory mouse model

Total volume of 25–30 μl of 0.1% λ-carrageenan (Sigma-Aldrich Corp., St. Louis, Missouri, USA) dissolved in saline (at 60^o^ C) and cooled further, was subcutaneously injected in the mice through plantar surface of left hind paw.

### Pain behavior tests

Thermal pain behaviors (Hargreaves) were assessed as previously described [6]. Mice were distributed randomly into groups (N = 8, per group) and thermal behavioral tests were performed in a blinded manner. The baseline latencies were adjusted to 5–7 seconds with 15 seconds taken as cutoff threshold to prevent potential injury. The latencies were averaged over 5 trials, separated by a 10-min interval.

#### Immunohistochemistry (IHC)

HEK293 (ATCC) and NBL (Neuroblastoma, ATCC) cells were plated in 12-well plates with cover slips coated prior with poly-D-lysine, laminin-coated glass coverslips for immunofluorescence. Cells were seeded at a density of 1x 10^5^ cells/ml. Culture volume was 2ml per well in 6-well plates, 1ml per well in 12-well plates. The cultures were incubated at 37°C in a water saturated atmosphere containing 5% CO_2_/95% air and maintained in Gibco DMEM+F12 (Invitrogen) supplemented with 10% FBS (Invitrogen) and 1X penicillin-streptomycin (Fisher Scientific, Pittsburgh, PA). Transfections were performed using AAV8 vectors containing tagged genes of interest (CA8-201^MT^, CA8-201, CA8-204^G^, CA8-204^C^).

Mice were anesthetized with isoflurane and perfused by a saline injection through the left ventricle with saline (30ml) followed by a thorough perfusion with a fixative of 4% paraformaldehyde+15% saturated picric acid solution in 0.16M PBS (pH~7.4, 4°C). Dorsal horn (DH) and DRG from the fourth and fifth lumbar regions (L4-L5) were dissected, and placed in the same fixative for 2–4 h post fix, then transferred to 20% sucrose overnight, ideally when the tissue touched the bottom. The tissues were embedded with OCT (Andwin Scientific Inc, Schaumburg, IL) on dry ice. From the embedded tissues, 16–20μm sections were cut using Leica 1900 Cryostat (Leica Microsystems Inc., Buffalo Grove, IL), and mounted on the slides for immunofluorescence staining.

The immunostaining of DRG sections was performed essentially as previously described procedure [5, 6]. Briefly, slides with DRG sections were washed in 1XPBS directly. Cell cultures on coated cover slips were washed twice with PBS and then fixed by 4% PFA in PBS for 15–30 min. Cell cultures and DRG sections on slides were blocked in 4% normal serum with 0.3% triton X-100 (Jackson ImmunoResearch Laboratories, West Grove, PA) for 60 min. Primary antibodies specific for V5 (Abcam, Cambridge, MA), FLAG (Aves labs, OR), Advillin (Invitrogen), S100 (Dako, Agilent) were diluted in PBS according to the instructions with 0.1% Triton X-100 and allowed to incubate with sections and cell cultures overnight at 4°C. Sections and cultures were washed 3X for 10 min each in 1XPBS, followed by incubation in either Alexa Fluor 488/ Alexa Fluor 594-conjugated secondary antibodies (Invitrogen) for 60 min at room temperature. The sections and cultures were washed again 3X for 10 min in PBS and counter stained with DAPI (Invitrogen). For cover slips with cultures and slides were mounted with 40mm cover slips with a gel/mount anti-fading mounting media (Biomeda, Foster City, CA). DAPI (Sigma) was mixed with secondary antibody for counting the total number of cells. Immunofluorescence images were acquired using an inverted microscope (DMI 6000 B, Leica, Germany). Advillin staining was used for total neuron counting while S100 was used to count total glial cell counts. Gamma, gain and exposure levels were set for control sections and kept constant for all other test sections.

### Data analyses

Analyses of data from immune staining of CA8-201^MT^, CA8-201 and CA8-204^G^ in DRG and the percentage of overexpression of positive neurons and glial cells in L4 and L5 DRG sections from nonadjacent slices (N = 8) from each group of animals were determined as described previously [6]. Percentages were calculated from at least 4 sections from at least 8 mice in each group and were averaged to calculate marker overexpression and overlap. In order to quantify immune-reactive double-staining in DRG, markers for glial cells (S100) and neurons (advillin) were divided by the total number of positive FLAG (for CA8-204^G^) and V5 (for CA8-201^MT^ and CA8-201) cells and for each occurrence. The investigator was blinded to the group and arrangements of ganglia on the slides while making a quantitative assessment. Data were summarized using mean ± SEM. Differences between groups were assessed using one-way / two-way ANOVA depending on the condition analyzed. The criterion for statistical significance was P<0.05 applying Bonferroni’s correction for multiple tests.

Quantitation of data in all the other experiments was performed using one-way ANOVA followed by Bonferroni correction / Tukey’s multiple group comparison as post-hoc test for experiments with triplicates or more, with experiments repeated 3 times or more. Quantitation of data on thermal pain behavior tests was performed using two-way ANOVA repeated measures for each time-point (day) the measurements were recorded, followed by Bonferroni’s post-hoc correction.

## Supporting information

S1 Fig CA8-204^G^ demonstrates a differential expression in neuronal and non-neuronal derived cell lines. **(I) (A)** Immunostaining (IF) using anti-V5 (V5- CA8-201^MT^, green) antibodies. **(B)** IF using antibodies anti-V5 (V5-CA8-201, green). **(C)** IF using anti-FLAG (FLAG-CA8-204^G^, green). IF from the HEK-293 cells transfected with AAV8-viral vectors containing CA8-201^MT^, CA8-201 and CA8-204^G^ shows an increase in positive overexpression in CA8-201 and CA8-204^G^. This expression was not detected in the CA8-201^MT^ in HEK-293 cells. **(II) (A)** IF using anti-V5 (V5-CA8-201^MT^, green) antibodies. **(B)** IF using anti-V5 antibodies (V5-CA8-201, green). **(C)** IF using anti-FLAG antibodies (CA8-204^G^, green). IF from NBL cells transfected with AAV8 virus containing CA8-201^MT^, CA8-201, or CA8-204^G^ showed an increase CA8-201 overexpression only. V5 expression was barely detectable after transfection with CA8-201^MT^ or CA8-204^G^ in NBL cells. **(III)** The histogram represents the comparison of overexpression of CA8-204 with CA8-201, where percentages of overexpression were quantified using one-way ANOVA with Bonferroni's post-hoc test (****P<0.0001, ***P<0.001, N = 8 each gene group). Comparisons reveal the ubiquitous nature of CA8-201 overexpression in both HEK and NBL cells. Exogenous expression of CA8-204^G^ was higher in HEK cells than NBL cells. There was virtually no V5-CA8 expression observed after transfection with CA8-201^MT^ in either cell line. (Scale: 50μm).
(TIF)

S2 Fig CA8-204 is unable to inhibit the activation of ITPR1 by phosphorylation (pITPR1) after NBL cell transfections. Immunoblot of pITPR1 (forskolin-induced phosphorylation; 10μM forskolin) in NBL cells, transfected with an empty vector, CA8-201 (WT) or CA8-204^G^. Western blotting suggests that while inhibition of pITPR1 expression through CA8-201 is observed, CA8-204^G^ was unable to inhibit pITPR1expression in NBL cells due to absence of any CA8-204^G^ expression. Quantitation of pITPR1 expression was performed using ImageJ software. Data were normalized with vinculin. N = 3, ***P<0.001, **P<0.01 Quantitative analysis was performed using the one-way ANOVA followed by Bonferroni post–hoc test for each possible comparison (GraphPad software).
(TIF)

S3 Fig CA8-201^MT^ fails to be expressed in either glial or neuronal cells. Immunostaining of cells extracted from DRG of mice that received sciatic nerve injections of AAV8-V5-CA8-201^MT^, used in this study as a negative control, were stained with **(A)** glial (S100) or neuronal (advillin) markers. (A) IF done with anti-V5 against V5-CA8-201^MT^ and anti-S100 (glial marker) antibodies individually in DRG sections, (V5, green; S100, red), and shown as merged (Merge). **(B)** IF done with anti-V5 and anti-advillin antibodies individually in DRG sections, (V5, green; advillin, red), and shown as merged (Merge). V5-CA8-201^MT^ failed to express in either glial or neuronal DRG cells. (Scale: 50μm).
(TIF)

S4 Fig Primary antibodies for anti-FLAG and anti-V5 omitted and secondary fluorescent conjugated antibodies (Alexa Fluor 488 and Alexa Fluor 594) were used as negative controls in DRG sections containing AAV8 viral vectors carrying V5-CA8-201 and FLAG-CA8-204^G^, respectively (Scale: 50μm). (TIF)
