# Supplementary figures and images for: Profound analgesia is associated with a truncated peptide resulting from tissue specific alternative splicing of DRG CA8-204 regulated by an exon-level cis-eQTL
Source: PLoS Genet. 2019 Jun 14;15(6):e1008226. doi: 10.1371/journal.pgen.1008226 (PMC6615631; doi:10.1371/journal.pgen.1008226)

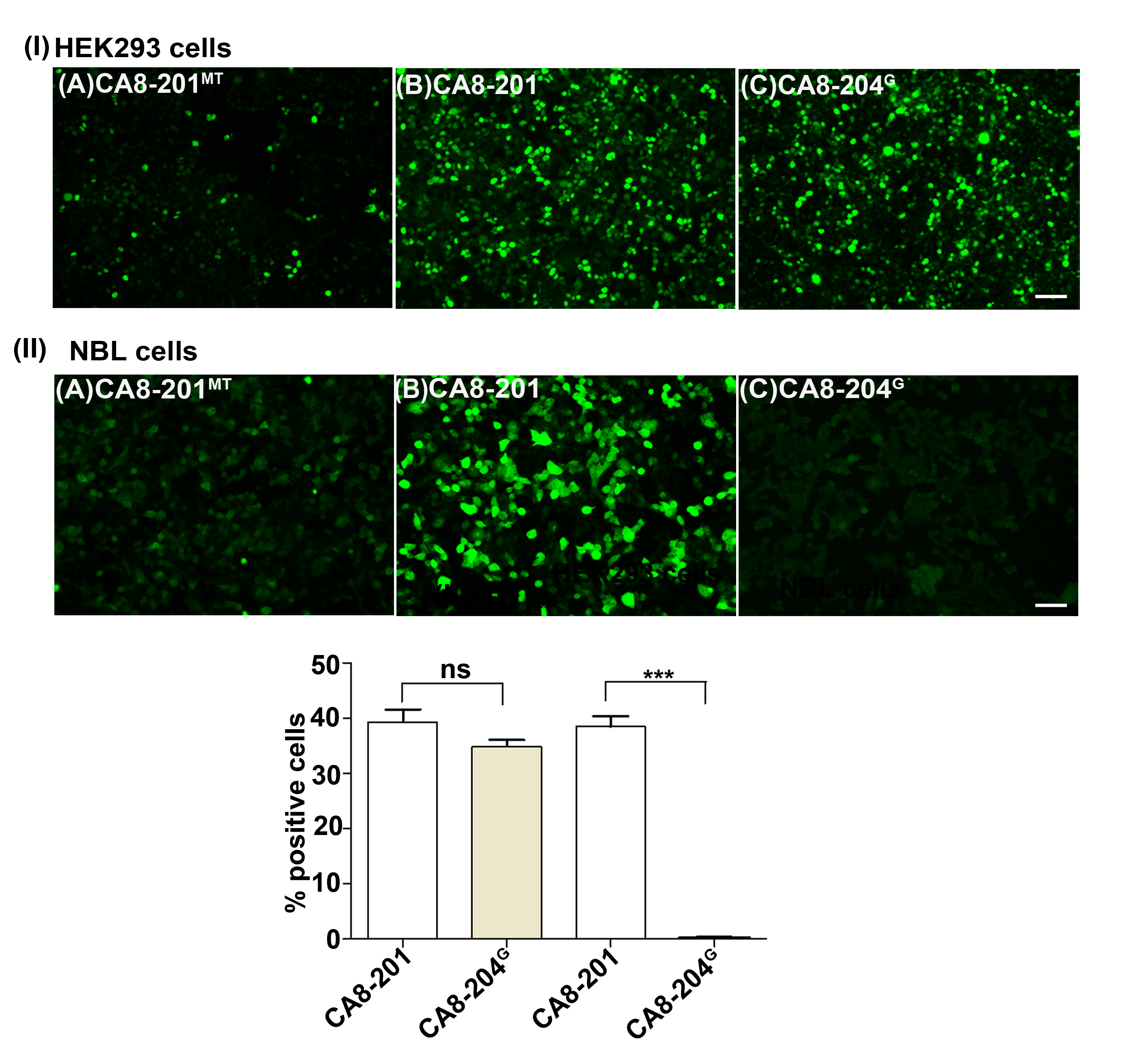

Supplement: S1 Fig — (I) (A) Immunostaining (IF) using anti-V5 (V5- CA8-201MT, green) antibodies. (B) IF using antibodies anti-V5 (V5-CA8-201, green). (C) IF using anti-FLAG (FLAG-CA8-204G, green). IF from the HEK-293 cells transfected with AAV8-viral vectors containing CA8-201MT, CA8-201 and CA8-204G shows an increase in positive overexpression in CA8-201 and CA8-204G. This expression was not detected in the CA8-201MT in HEK-293 cells. (II) (A) IF using anti-V5 (V5-CA8-201MT, green) antibodies. (B) IF using anti-V5 antibodies (V5-CA8-201, green). (C) IF using anti-FLAG antibodies (CA8-204G, green). IF from NBL cells transfected with AAV8 virus containing CA8-201MT, CA8-201, or CA8-204G showed an increase CA8-201 overexpression only. V5 expression was barely detectable after transfection with CA8-201MT or CA8-204G in NBL cells. (III) The histogram represents the comparison of overexpression of CA8-204 with CA8-201, where percentages of overexpression were quantified using one-way ANOVA with Bonferroni's post-hoc test (****P<0.0001, ***P<0.001, N = 8 each gene group). Comparisons reveal the ubiquitous nature of CA8-201 overexpression in both HEK and NBL cells. Exogenous expression of CA8-204G was higher in HEK cells than NBL cells. There was virtually no V5-CA8 expression observed after transfection with CA8-201MT in either cell line. (Scale: 50μm). (TIF) [file pgen.1008226.s001.tif]

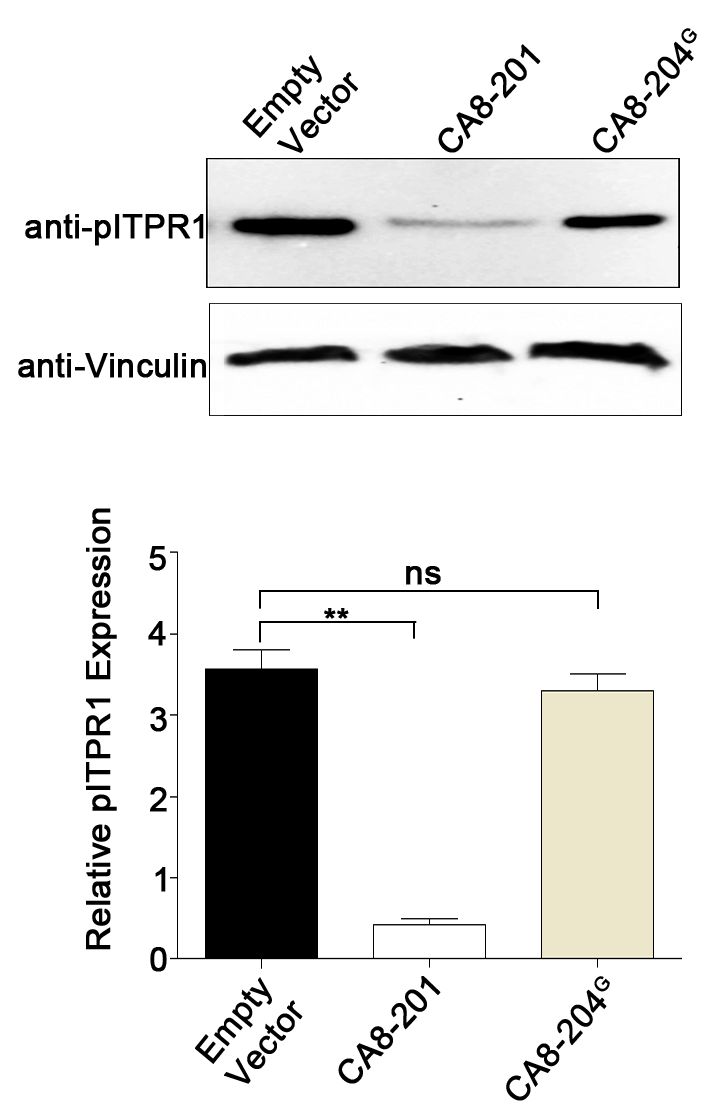

Supplement: S2 Fig — Immunoblot of pITPR1 (forskolin-induced phosphorylation; 10μM forskolin) in NBL cells, transfected with an empty vector, CA8-201 (WT) or CA8-204G. Western blotting suggests that while inhibition of pITPR1 expression through CA8-201 is observed, CA8-204G was unable to inhibit pITPR1expression in NBL cells due to absence of any CA8-204G expression. Quantitation of pITPR1 expression was performed using ImageJ software. Data were normalized with vinculin. N = 3, ***P<0.001, **P<0.01 Quantitative analysis was performed using the one-way ANOVA followed by Bonferroni post–hoc test for each possible comparison (GraphPad software). (TIF) [file pgen.1008226.s002.tif]

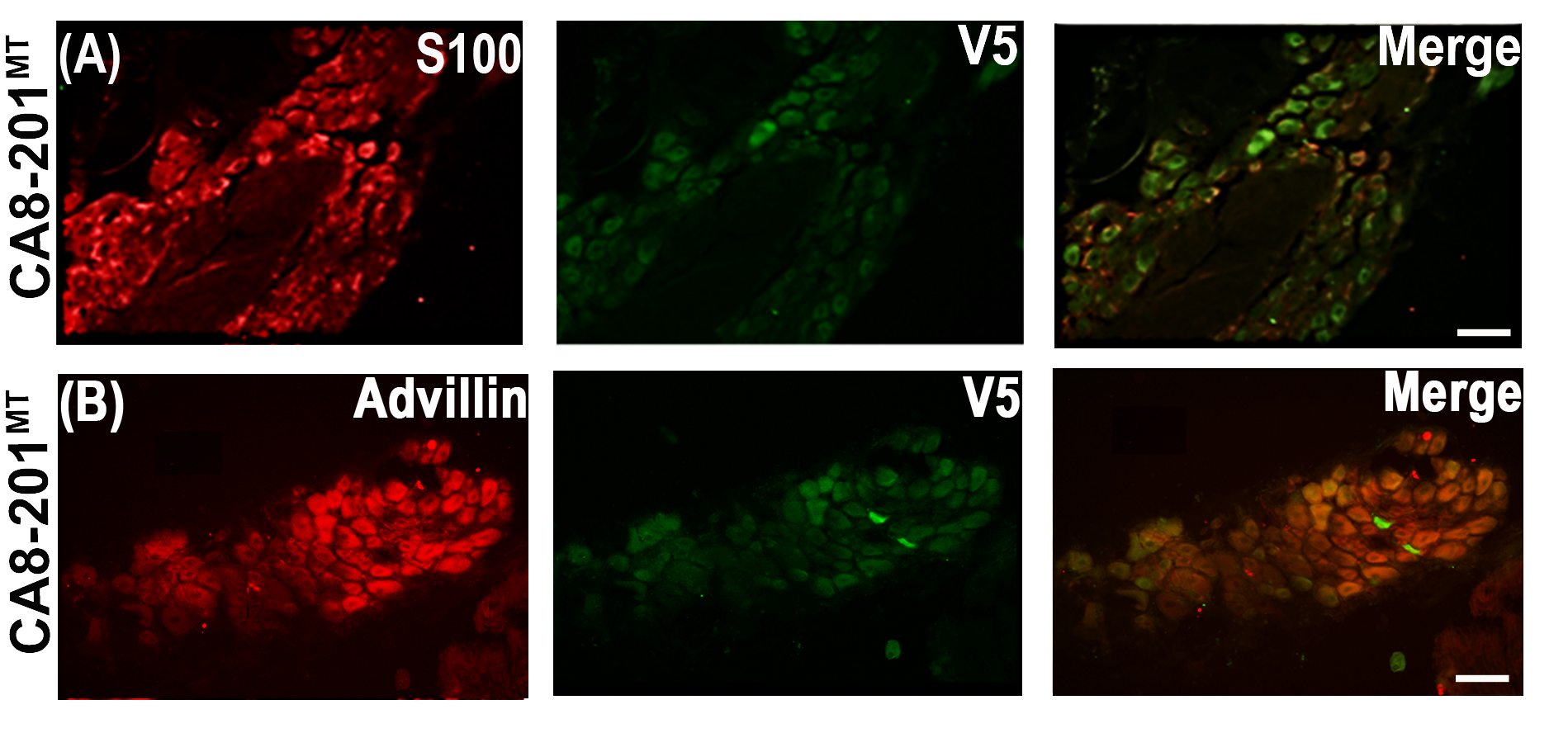

Supplement: S3 Fig — Immunostaining of cells extracted from DRG of mice that received sciatic nerve injections of AAV8-V5-CA8-201MT, used in this study as a negative control, were stained with (A) glial (S100) or neuronal (advillin) markers. (A) IF done with anti-V5 against V5-CA8-201MT and anti-S100 (glial marker) antibodies individually in DRG sections, (V5, green; S100, red), and shown as merged (Merge). (B) IF done with anti-V5 and anti-advillin antibodies individually in DRG sections, (V5, green; advillin, red), and shown as merged (Merge). V5-CA8-201MT failed to express in either glial or neuronal DRG cells. (Scale: 50μm). (TIF) [file pgen.1008226.s003.tif]

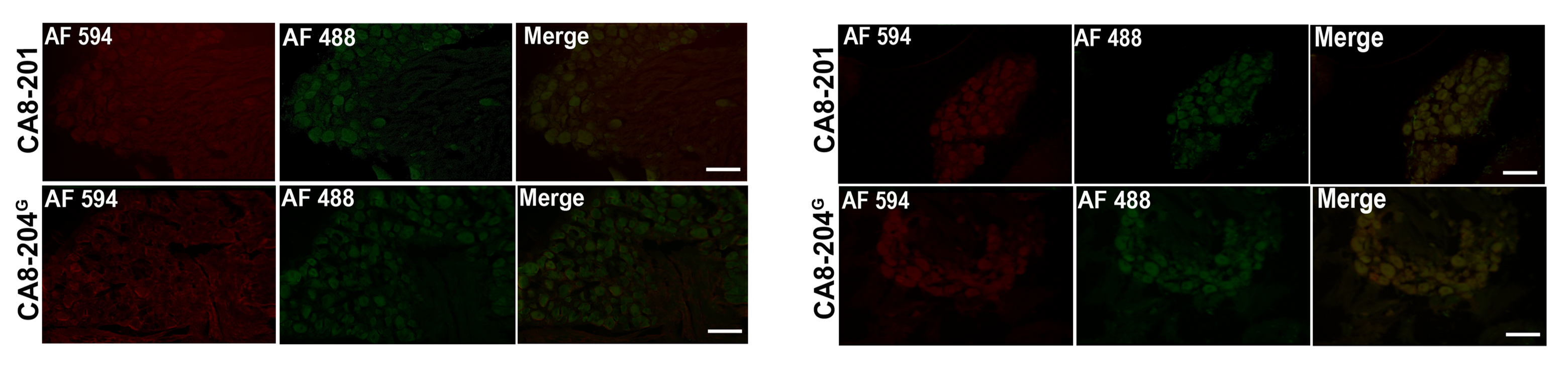

Supplement: S4 Fig — (TIF) [file pgen.1008226.s004.tif]
